# Current Diagnostic, Counseling, and Treatment Options in Non-Severe and Severe Apparently Isolated Fetal Ventriculomegaly

**DOI:** 10.3390/biomedicines12122929

**Published:** 2024-12-23

**Authors:** Mateusz Zamłyński, Marta Grokhovska, Andrea Surányi, Anita Olejek

**Affiliations:** 1 Department of Gynecology, Obstetrics and Oncological Gynecology, Faculty of Medical Sciences in Zabrze, Medical University of Silesia, Stefana Batorego 15, 41-902 Bytom, Poland; anitaolejek@wp.pl; 2 Department of Obstetrics, Gynecology and Perinatology, Lviv National Medical University of Danylo Halytskyy, Pekarska 69, 79010 Lviv, Ukraine; marta.grokhovska@ukr.net; 3 Department of Obstetrics & Gynecology, Albert Szent-Györgyi Medical School, University of Szeged, 1 Semmelweis Str., 6720 Szeged, Hungary; gaspar-suranyi.andrea@med.u-szeged.hu

**Keywords:** isolated fetal ventriculomegaly, ventriculomegaly, fetal therapy, ventriculomegaly treatment

## Abstract

The widening of the vestibular dimension of lateral ventricles > 10 mm should be considered a symptom rather than a definitive diagnosis. In fact, fetal ventriculomegaly (VM) is a defect with ’multifaceted‘ clinical consequences in the child’s further neurodevelopment. Isolated fetal ventriculomegaly can cause neurological defects ranging from mild neurodevelopmental delay to severe complications in the form of ongoing palliative care to the death of patients at various developmental periods. The spectrum of compilations often depends on the severity of the ventriculomegaly. In the prenatal period, the combined diagnostic tools include the following: ultrasound/MRI and genetic, infectious tests that form the basis of reliable counseling. We hypothesize that advances in the diagnostic process allow the identification of ‘probably’ isolated forms of severe VM (ISVM). The review authors electronically searched MEDLINE, EMBASE, and the Cochrane Library databases, describing the evidence-based validity and option of prenatal decompression for ISVM. The purpose of this review is to present the evolution of diagnostic techniques and views indicating the possibility and limitations of implementing prenatal decompression in severe ISVM. In conclusion, after reviewing the available data, we want to introduce the idea that perinatal centers are close to or have reached the necessary capability, expertise, and competence to perform ISVM decompression procedures. Endoscopic ventriculostomy of the third ventricle (ETV) appears to be promising, as it seems to be associated with minimal perinatal complications and better neurological outcomes for the newborn. However, long-term follow-up results for the neurodevelopment of patients who underwent ETV have not been reported. Looking ahead, randomized trials with the long-term neurodevelopmental follow-up of children who underwent prenatal decompression due to ISVM are needed.

## 1. Introduction

Fetal ventriculomegaly (VM) is a general term used to describe the enlargement of the cerebral ventricles, diagnosed in utero, with the estimated prevalence of 1–2 in 1000 births [1]. It is one of the most common fetal anomalies detected on ultrasound (US) between 18 and 24 weeks of gestational age (GA) [1]. VM is the most frequently observed (23.36%) malformation of the central nervous system (CNS), with over half of the fetuses with VM presenting with associated extra-CNS anomalies [2]. Non-isolated VM is a heterogeneous disease, with multiple intracranial and extracranial etiologies and a broad spectrum of poor neurological outcomes, often secondary to the main defect. The prognosis is associated with the primary diagnosis, such as hydranencephaly, schizencephaly, the Chiari malformation, or the Dandy–Walker syndrome [3]. The prevalence of severe ventriculomegaly (SVM) at birth has been estimated at 0.3–1.5 in 1000 gestations [4]. Apparently isolated severe ventriculomegaly is extremely rare—two in ten-thousand births [4].

The width of the lateral ventricles is determined using the standardized atrial diameter (AD), where the temporal and frontal horns of the lateral ventricles converge [5]. In prenatal diagnostics, VM is classified as mild (AD of 10–12 mm), moderate (AD of 13–15 mm), and severe (AD > 15 mm) [5]. In order to improve the clarity of neurodevelopmental nomenclature and counseling of the parents of the affected children, the terms ‘isolated non-severe ventriculomegaly (INSVM) and ‘isolated severe ventriculomegaly’ (ISVM) are used for mild and moderate isolated VM, respectively [6]. The pathogenesis of various types of VM presents a 5% risk for aneuploidy (mainly trisomy 21), 10–15% for other genetic defects, 5% for congenital infections such as cytomegaly, toxoplasmosis, and zika virus [7]. Therefore, it is necessary to use dedicated diagnostic tools to identify the ‘most probable’ diagnosis, counseling, and therapeutic options for INSVM and ISVM.

## 2. INSVM and ISVM Fetal Diagnostic Tools

### 2.1. Imaging by US

**Figure 1:**
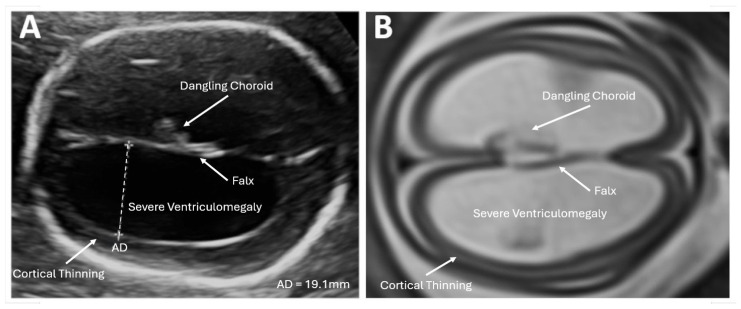
Imaging in the transventricular plane of ISVM at 22.3 weeks of pregnancy: (**A**) transabdominal ultrasound: the atrial width of the distal ventricle is increased at AD = 19.1 mm, and (**B**) MRI of T2-dependent sequence SSFSE.

**Figure 2:**
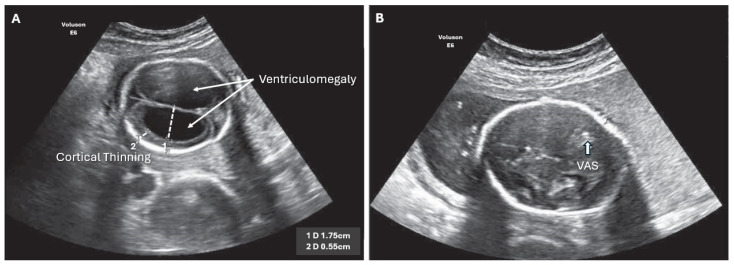
ISVM fetal ultrasound before (**A**,**B**) after VAS implantation, arrow indicates VAS.

Sonographic evaluation remains the imaging test of choice due to its ease of application, test repeatability, low cost, and availability of the equipment at fetal medicine centers [8]. The standardized AD should be measured in the transventricular plane (axial) at the level that demonstrates the frontal horns and cavum septi pellucidi, in which the appearance of the hemispheres is symmetrical [9]. The caliper should be placed on the interior margin of the medial and lateral wall of the atria, at the level of parietal-occipital groove and glomus of the choroid plexus, on the axis that is perpendicular to the long axis of the left ventricle (Figure 1 and Figure 2). The measurements are perpendicular to the long axis of the left ventricle. In the case of fetal VM, the choroid plexus appears to be ‘dangling’ or falling towards the dependent ventricular wall and occupies less space in the ventricle than in cases without VM [5]. In cases without VM, the choroid plexus typically takes up at least half or more of the lateral ventricle [10]. Asymmetric VM is defined in the second trimester of pregnancy as an AD difference of >2 mm, with one or both ventricles larger than >10 mm [11]. Unilateral dilation of the ventricles is reported for 50–60% of the cases [5]. Patients with unilateral VM have similar neurodevelopmental outcomes as those with bilateral VM [12]. Agenesis of the corpus collosum (ACC), which is caused by the blockage of that structure by the dilated VM, is often observed in INSVM as well as ISVM. Color Doppler ultrasound is useful when searching for the periventricular aorta to confirm complete or partial agenesis of the corpus collosum [13].

Isolated non-severe ventriculomegaly (INSVM) is diagnosed on ultrasound for an AD of >10–14.9 mm and in the absence of other associated cranial and non-cranial malformations, together with the echocardiography of the fetal heart [6,14]. As further diagnostic procedures are necessary, INSVM is the diagnosis of only the ‘apparent’ malformation [15,16]. The parents should be informed that it is merely a preliminary diagnosis, and that, in 13% of the cases, it is initially not possible to identify other serious cerebral defects. Further observation using repeated ultrasound testing is necessary as the AD value does not have a uniform model of evolution: stability—55%, regression—30%, and progression to the AD ≥ 15 mm in 16% of the cases [17,18]. Perlman et al. compared prenatal and postnatal imaging in a cohort of 92 INSVM fetuses (AD ≥ 10–<14 mm) and found statistically significant regression of the prenatal isolated ventriculomegaly (*p* < 001). During clinical follow-up at 24 months, normal development was observed in all infants except three, who presented with very mild neurological deficits [19].

Isolated severe ventriculomegaly (ISVM) is diagnosed on ultrasound for an AD of ≥15 mm [16]. Third ventricle imaging on the sagittal plane using 3D transvaginal neurosonography is the new measurement point for ISVM due to the fact that it reveals a strong correlation between the interthalamic adhesion diameter and postnatal diagnosis of aqueductal stenosis (98.6% accuracy, CI 0.92–0.99) [20,21]. Brinbaun et al. reported that transvaginal neurosonography allows visualization of the normal and abnormal third ventricle of the fetus. An ITAD < 7.1 identifies aqueductal stenosis as the likely etiology of severe ventriculomegaly with 98.6% accuracy [20].

As in INSVM, neurosonography in ISVM may visualize significant changes in the AD size, even over a brief period of time [21,22]. Isolated aqueductal stenosis (IAS), which is the result of non-communicating ventriculomegaly with a normally developed brain, is an example of the dynamics of AD change [23]. Ge et al., in a cohort of 36 fetuses with ISVM, used repeated neurosonography testing and determined the weekly ventricular expansion for two groups: ventricular acceleration of ≥3 mm/week as compared to a plateau of >3 mm/week [24]. The comparison of the mean ventricular progression of an AD ≥ 3 mm/week vs. >3 mm/week was 4.1 mm/week vs. 1.0 mm/week, respectively (*p* = 0.031). All patients with an increase of ≥3 mm/week required VP shunting [24]. Similarly, Kline-Fath et al., in their MRI study of 30 fetuses with aqueductal stenosis, found that progressive SVM was associated with an increase in ventricular rupture—60%, the loss of septal leaflets—47%, and a reduction in white matter and corpus collosum volume—43%. Cerebellar ectopia developed in 27% of the patients, and, out of those, 6% met the criteria for Chiari I malformation [25]. Postnatally, 97% of the infants required VP shunting. These authors determined that the AD increased in size from the time of prenatal diagnosis of SVM to the time of birth at a rate of 1.2 mm/week [25].

### 2.2. Imaging by MRI

Fetal magnetic resonance imaging (MRI) in spatial projections and multidirectional scanning in sagittal, frontal, and axial planes demonstrate contrast between tissues, offering superior resolution of the soft tissues, with a big field of view and the possibility of obtaining the full image of the fetal body [26]. An entire range of additional intracranial anomalies is diagnosed using traditional as well as advanced MRI. Contrast-enhanced T2-weighted imaging using turbo-field spin echo (SE) or steady state free-precession (SSFP) sequences remains the main imaging technique for the fetal CNS. Advanced MRI techniques include axial diffusion-weighted imaging (DWI) for the examination of brain tissue cellularity and water movement, which is useful when attempting to assess cerebral infarction or hemorrhage [26,27]. Additionally, echo planar imaging (EPI) increases the diagnostic performance for intracranial hemorrhage [27]. Axial T-1 imaging of the brain is used to detect intracranial lipoma, germinal matrix hemorrhage, or thrombosis [27]. The T2-dependent contrast technique using fast (turbo) spin-echo (SE) sequence is also recommended.

Heaphy-Henault et al. used MRI in 43 fetuses and described specific features of ISVM with IAS, which included the following: aqueduct funneling, enlarged recesses of the third ventricle, and an abnormal CC [28]. A significantly increased probability of IAS was associated with the following imaging findings: enlarged recesses of the third ventricle, aqueduct funneling, hemorrhage in the cerebral aqueduct, ventricular diverticulum, rhombencephalosynapsis, and dystroglycanopathy-related cerebellar dysplasia [28].

New MRI techniques allow for the evaluation of cortical development, both gray and white matter, in fetuses with INSVM. MRI in 41 fetuses with isolated VM (AD 10.0–12.0 mm) demonstrated the abnormal development of transient fetal brain zones belonging to both compartments, regardless of the VM-affected side [29]. In another study, Benkarim et al. used MRI images from 23 fetuses with INSVM and investigated cortical folding alterations and the effect of ipsilateral ventricular enlargement associated with decreased cortical folding [6]. Hahner et al., in their MRI study of 32 fetuses with INSVM, evaluated global and regional changes in the cortical development and reported decreased cortical volume in the frontal lobes, which were associated with neonatal neurobehavior [15]. In their latest study, Brady et al. reported that the third ventricle volume varies depending on gestational age but typically measures < 4 mm in the transverse dimension. A normal fourth ventricle does not differentiate between non-communicating and communicating hydrocephalus. Additionally, these authors suggested the possibility of applying artificial intelligence (AI) in MRI of the fetal brain, which would adjust for fetal motion artifacts that are the consequences of fetal movement in utero [30]. The integration of AI can affect the reduction in artifacts resulting from fetal movements as well as maternal breathing. The convolutional neural network (CNN) developed by Gagoski et al. automatically detects artifacts on T2 HASTE sequences during fetal MRI to improve image quality. This system selects images of the lowest quality, and only those are re-acquired, resulting in shorter examination time. Also noteworthy is DL (deep learning), which has been developing very intensively in recent years [31]. Xu et al. developed a DL-based system that, among other features, provides fetal motion tracking and may automate the readjustment of acquisition parameters. In less than 1 s, their model could predict fetal posture with an accuracy of 4.5 mm. Another study that uses AI appliances in fetal MRI is SVRnet (preprocessing AI system) [32]. The team of Hou developed a conceptual method by generating two-dimensional T2-weighted single slices in varying orientations to reduce the artifact made by its jerky movements. They produced qualitatively enhanced reconstructions and achieved a spatial prediction error of 7 mm on simulated data for moving fetuses at around 20 weeks of gestation. The 2D/3D registration initialization problem was solved in a broad and computationally efficient way, making it appropriate for usage in real-time settings. Similarly, Singh et al.’s group described in their manuscript the use of DL in correcting artifacts resulting from fetal movement. Their method derived from predicting fetal motion directly from acquired images in real time, using anatomical information extracted from slice sequences. The neural network could predict fetal motion within 8 degrees and estimate motion-affected slices to schedule subsequent collections [33].

### 2.3. Complementary Diagnosis of Fetal Ultrasound and MRI

Significant advancements in the field of US and MRI did not eliminate problems with image quality caused by the following factors: significant maternal weight, maternal breathing movements, abdominal scars, anterior placenta, oligohydramnios, non-cephalic fetal presentation, and fetal movement [5,16]. The advantages of US imaging in fetal ventriculomegaly include the repeatability of the test, accessibility, and repeated measurements of the AD value [14,34]. MRI is typically performed after repeated US neuroimaging at >19 weeks of GA, with the optimal diagnostic window at 22 weeks of GA, and constitutes an additional value that conclusively determines the type of VM [35,36].

Griffiths et al. demonstrated that MRI is characterized by higher sensitivity—as compared to US—improved counseling, and management in 22.2% of fetuses with different CNS anomalies [37]. According to the ‘Use of MRI to enhance the diagnosis of fetal developmental brain abnormalities in utero’ MERIDIAN study, US sensitivity and specificity in the diagnosis of fetal cerebral anomalies was 70% at <24 and 64% at >24 weeks GA. The decrease in the diagnostic value is caused by fetal presentation and ossification of the cranial vault. The MERIDIAN study included 570 cases and demonstrated that the rate of high-confidence diagnoses increased by 13% after MRI, from 82% to 95% [38]. Likewise, Barzilay et al., in their MRI investigation of 68 fetuses at 32 weeks of GA, found additional intracranial anomalies in the following cases: moderate—6.1% and ISVM—25%. The combined rate of additional findings in INSVM was 3.3% (95% CI: 0.9–11.4%) [39].

In cases of INSVM diagnosed by US, MRI may detect an additional 5% of fetal abnormalities [40]. Di Mascio et al. demonstrated that the rate of additional structural anomalies in ‘apparently’ isolated severe VM detected exclusively with fetal brain MRI was 18.1% [41]. Defects of the cerebral cortex and the midline included the most common types of cerebral anomalies. Additionally, ISVM was independently associated with a higher probability of detecting an anomaly on MRI. Finally, the rate of the associated anomalies detected only postnatally and undetected on prenatal imaging was 13.6% [42]. The efficiency of US or MRI in ISVM caused by IAS is comparable as far as the detection of the following CNS malformations is concerned: fCM, ACC, cerebellar anomalies, cerebral cortex anomalies, and absence of the cavum septum pellucidum [34,43].

Current 3DUS and MRI-3D-Slicer imaging techniques are used for the volumetric assessment of the four ventricular horns and the analysis of the developmental changes in the fetal brain. Knowledge about the developmental evolution of the shape and size of the lateral ventricular horns is vital to diagnose isolated VM and may improve prenatal counseling, management strategies, pregnancy outcomes, and further neurological status [44].

Emery et al., in the North American Fetal Therapy Network (NAFTNet) multicenter, prospective observational study of 32 fetuses with AS with a postnatally confirmed diagnosis, pointed out that high-resolution US identifies features of SVM: midline symmetry, thinning of the frontal, parietal, and occipital cortex, overhanging choroid, dilated third ventricle, and normal posterior cranial fossa. In addition, MRI can diagnose the loss of off-axis space and occlusion of the Sylvian aqueduct between the third and fourth ventricles [45].

Combined findings of the US/MRI improve accuracy of the prognosis about the development of the affected child reported during counseling.

#### Imaging Tools Redefined

Ultrasound serial measurements of a fetal AD can, in a limited way, be considered an indicator of increased intracranial pressure. However, the presence of ventricular enlargement on fetal imaging does not always correlate with postnatal hydrocephalus and does not provide information about any effect on neural tissue and biochemical pathways [46]. In many cases, by the time significant VM has occurred or even without significant ventricular enlargement, irreversible brain tissue damage has already occurred [47].

An important advance in MR imaging techniques is the ability to determine the neuro-metabolic status of the fetal brain with SVM using MRI-based oxygen extraction fraction mapping [48]. A study of Zhuang et al., described that, in adult humans with normotensive hydrocephalus, the oxygen extraction fraction was significantly lower than in the healthy individuals in the control group, and this was observed both in the whole brain and in specific regions (gray matter, thalamus, caudate and mantle) [48]. Mapping oxygen extraction in VM fetuses is most certainly a matter of the near future.

The development of volumetric MR techniques also provides valuable information regarding the structure of the CNS ventricular system and the dependent metabolic state of the brain [49]. Ebner et al. developed isotropic motion-corrected volumetric reconstruction based on acquired 2D image stacks, including the so-called super-resolution reconstruction method [50]. In a new study, Deprest et al. demonstrated the feasibility of obtaining volumetric images of the fetal brain using T2-weighted 2D slices that were converted to 3D volumes using a super-resolution reconstruction algorithm. The resulting volumetric data were then processed by a new convolutional neural network, which enabled segmentation of the white matter, ventricular system, and cerebellum [51].

The use of brain elastography of newborns with VM has shown that the ARFI acoustic radiation force pulse determines increased ‘stiffness’, which suggests that additional changes in brain tissue are being detected [52]. The studies of fetal brain elastography are in the experimental research area.

Current Doppler techniques for diagnosing intracranial ICP pressure are focused on measuring flow resistance parameters in the anterior cerebral artery. The results obtained do not definitively represent the actual ICP because this value depends on extracranial factors [53].

The new MV-FlowTM and Lumi FlowTM (Samsung Medison Co. Ltd., Seoul, South Korea) Doppler technologies provide an alternative to Power Doppler for visualizing slow-flowing microvascular structures and vascular connections [54].

### 2.4. Genetic Diagnosis Defining Apparently Isolated Fetal Vm

Genetic testing constitutes the basis for the diagnosis and team consultation on prenatal management and the postnatal prognosis [55]. The diagnosis of apparently isolated VM can be made only if a normal karyotype is confirmed [11,56]. Chorionic villous sampling or amniocentesis in VM should include DNA copy number variants detected with microarray (aCGH) genotyping for defects associated with common aneuploidies of chromosomes 13, 18, 21 XY, with possible findings of microdeletions and microduplications—6.7% of the cases [57,58].

According to Toren et al., the rate of CGH microarray aberrations in non-isolated VM cases is 24.1% as compared to 6.2% for ISVM (*p* = 0.031) [59]. The rate of pathologic karyotypes in IVM is lower than in cases when VM presents with additional structural defects (1.5–12% vs. 9.5–36%, respectively) [60]. The prevalence of genetic aberrations is not influenced by the severity of the VM dilation [60].

Recent years have witnessed the identification of over 100 genes associated with fetal VM, in most cases, as part of a defined genetic syndrome [60]. Groups of multiorgan disorders linked with VM include the following: ciliopathies: Meckel syndrome, Gruber’s occipital encephalocele, Joubert’s encephalocele, Vermian syndrome; TUBA1A tubulinopathy: cortical developmental malformations, the dysgenesis of basal ganglia, agenesis/dysgenesis of the corpus callosum, cerebellar dysgenesis, midbrain abnormalities; and dystroglycanopathies: Walker–Warburg cortical developmental malformations, occipital cephalocele, agenesis/dysgenesis of the corpus callosum, a Z-shaped brainstem, and ocular malformations whose diagnosis requires non-standard approaches like exome sequencing (ES), with the possibility of detecting genetic abnormalities ranging from 19% to 44% [61,62,63].

Whole exome sequencing (WES) or whole genome sequencing (WGS) is the ultimate approach to genetic diagnostics for CNS defects [64]. In the Population Architecture through Genomics and Environment (PAGE) study, a total of 610 fetuses with structural anomalies were analyzed by WES, and a diagnostic genetic variant was identified only in 8.5% of the fetuses with an isolated cerebral anomaly, which is lower than suggested by earlier smaller-scale studies [64]. Imaging in a VM fetus may reveal agenesis of the corpus callosum (ACC). Monogenic mutations found in cases with ACC are typically associated with neurodevelopmental disorders secondary to *EPG5*, *ZEB2*, *SLC12A6*, and *AIC* gene mutations [65]. Mutations in the DCC Netrin 1 receptor gene were identified in isolated ACC, which is responsible for minor neurological symptoms and normal developmental outcomes, independently concomitant with VM [66].

There is no doubt that rapid advancements in ES technology will enhance our understanding of the primary mechanisms and associations of fetal VM, which in turn will help physicians supply the future parents with more meaningful antenatal information about their child [60]. However, for now, the available data about fetal phenotype–genotype correlations are limited. That is why advanced genome testing should be offered only by specialist referral centers that are adequately equipped and have the necessary experience [67].

The most prevalent form of heritable hydrocephalus is the X-linked hydrocephalus with the stenosis of the aqueduct of Sylvius, known as the L1 syndrome, caused by mutations in the *L1CAM* gene [68,69]. Mutations in the encoding gene *L1CAM* (L1 cell adhesion molecule) in *Xq28* are the main genetic causes of X-linked VM [69]. Mutations in *L1CAM* cause several structural and functional changes in the *L1CAM* protein and are responsible for obstruction in the CSF flow, mainly at the level of the aqueduct of Sylvius [70].

Other mutations associated with congenital hydrocephalus include multiple-PDZ domain protein-1 (MUPP-1), a tight junction protein, and CCDC88C (DAPLE) mutations of the Wnt signaling pathway [68]. Due to the unclear associations between the exome and the phenotype, the need for ES in prenatal development remains the topic of much debate, and further research is necessary [71].

### 2.5. Ivm Infectious Agent Testing

The prevalence of intraamniotic infection as the causative factor for fetal ventriculomegaly has been estimated at 1.4% [72]. Environmental factors, such as infections in the early stages of the fetal life, alter the structure of the fetal brain and become risk factors for neurodevelopmental disorders [73]. Infections lead to ventricular occlusion and abnormal development, which are examples of etiological risk factors for fetal VM [74]. Li et al., in their study of maternal serum from 70 pregnant women, demonstrated that elevated WBC and NE may be indicative of a perinatal infection as the cause of fetal VM [75]. The findings of their study should be taken into consideration in cases of idiopathic VM.

Cytomegalovirus and toxoplasmosis remain the most common infectious agents concomitant with VM [76]. Viral infections like *rubella*, *B19 parvovirus*, *zika virus*, and *herpes* may cause ventriculomegaly; therefore, women from high-risk groups should be tested [77]. Standard maternal TORCH serology for IgG and IgM, if positive and concomitant with VM or other developmental anomalies, is an indication for polymerase chain reaction (PCR) analysis of the amniotic fluid (AF) [78]. Maternal antibody serums or amniotic fluid avidity tests are conclusive in determining the activity of infectious agents only if the mother does not consent to the diagnostic technique of amniocentesis [78].

## 3. Counseling in IVM

Parent counseling for fetal IVM in the prenatal developmental stage signals the final phase of the diagnostic process. Counseling should integrate familial and current medical history, as well as the findings of repeated neurosonography, MRI, genetic testing, and infectious agent testing. The time and scope of counseling should not be limited in any way. Non-directive counseling is the optimal method of approaching the parents. It is good practice to include several members of the family who might support the parents in caring for an infant with IVM. The counseling team should consist of a perinatologist, fetal neurologist/pediatrician, and psychologist. If needed, specialists from the fields of fetal neurosurgery, orthopedics, and rehabilitation might also be included [42,79]. The scope of counseling should include the diagnostic findings for IVM, based on the current results of the neurodevelopmental tests.

### Outcomes in Fetuses with Isolated Ventriculomegaly

The main results in INSVM are as follows: AD (10.0-14.9 mm), survival rate (95–97%), risk for fetal demise (2%), and infant mortality (1.6%) [34,80].

Contemporary studies classify mild ventriculomegaly (IVM)—with an AD of <12 mm and a normal head circumference of >50th centile—as a normal developmental variant, which requires further imaging as the pregnancy progresses [16,81]. In mild IVM, risk factors due to infection were postnatally found in 7–10% of the children [16]. Possible additional malformations include malformations of the cortical development (MCDs) and corpus callosum (CC). During pregnancy, the following may be expected: 57.9%—resolution in utero, 26.3%—stability, and 15.8%—progression [7,18]. The resolution of INSVM in utero may not eliminate the risk for genetic or chromosomal abnormalities so advanced genetic testing—ES or WES—is advised [16]. The mode of delivery is based on the standard obstetric protocol.

In the case of mild IVM, parents should be made aware that a favorable neurodevelopmental outcome is observed in 90% of the affected children, which is close to that of the general population. After additional testing during infancy, they should be counseled that the child will more likely develop normally [82].

As far as moderate IVM (AD of 13–14.9 mm) is concerned, a normal postnatal AD is found in 75–93% of the fetuses [34,83]. Repeated follow-up ultrasound tests are necessary because progression is observed in 16% of the cases; the proof of progression may change both the diagnosis and the prognosis [83]. It has been demonstrated that the in utero progression of ventricular dilation is associated with neurological deficits in 44% of the newborns as compared to 7% in the non-progression group (RR, 6.32; 95% CI: 2.56–15.35%; *p* < 0.001) [83]. According to the current guidelines of the ISUOG Clinical Standards Committee, additional abnormalities found on ultrasound are not confirmed on MRI for the following: hemorrhagic lesions and parenchymal damage—75–93%, MCD—79–91%, partial CC abnormalities—63%, infratentorial abnormalities—63–100%, and vascular pathologies—44–68% [84].

The probability of postnatal neurosurgical intervention for INSVM is low [34].

The main results in ISVM are as follows: AD ≥ 15 mm; survival rate (30–40%), risk for fetal demise (12.1–24.3%), infant mortality (17.1%), and survival rate for the survivors (87.9%) [80,85].

Postnatally, additional abnormalities were found on MRI in 23.5% and 13.5% of cases with mild and severe developmental delay, respectively [85]. In a metanalysis of the outcomes of 41 survivors with ISVM, no disability was reported in 42.2%, mild/moderate disability in 18.6%, and severe disability in 39.6% of the cases [85]. Additional malformations, such as in moderate INSVM, have also been reported [84]. A meta-analysis by Ali et al. indicated that, among 249 cases of mild IVM, there were 21 (8.43%) cases of neurodevelopmental delay. In contrast, 37 (45.1%) cases of neurodevelopmental delay occurred among 82 cases of severe IVM. The risk of developmental delay is twice as high in patients with ISVM vs. INSVM, which was 4.24 [95% CI: 2.46–7.30%] [80]. Gomez-Arriaga et al. pointed out that the most common neurodevelopmental disabilities, specifically in severe IVM patients, are the gross motor skills, personal–social, and behavior domains. In long-term support planning, neurodevelopmental outcomes may be assessed using the Battelle Development Inventory (BDI). This tool can be used as early as 6 months of infancy. Although the sensitivity of the extended BDI is high and helps to avoid the risk of overlooking slight deficits, it may also falsely lead to overdiagnosis. Therefore, the clinical significance of this information should be established with the aid of an expert like a neuropediatrician and neuropsychologist [18].

Postnatal neurosurgical intervention in ISVM is probable in all patients with a dynamic AD increase during pregnancy [24].

In countries with a permissive policy for TOP due to unfavorable neurological prognosis (ISVM), there is an ethical obligation to inform the parents about such an option. In perinatal centers that offer multidisciplinary counseling for pregnant women, the rate for TOP has been estimated at 12–18% [79]. Start et al. demonstrated no association between the TOP decision and the AD of <20 mm and >20 mm (respectively, 24.4% vs. 22.2% *p* = 0.6) [86]. Tan et al. reported that 62.38% of the parents opt for TOP if the CNS anomalies had been diagnosed before 24 weeks of gestation. If the diagnosis is established after 24 weeks of gestation, 84.68% of the parents will choose to continue with the pregnancy [2]. Very late termination of pregnancy (VLTOP) at >24 weeks of gestation is inevitable in the case of delayed counseling caused by delayed diagnosis and may affect as many as 59% of the fetuses. VLTOP remains the topic of much heated ethical debate as TOP after 24 weeks of gestation is not acceptable in many countries [2].

## 4. Prenatal Treatment Options for ISVM

Ventriculomegaly is defined as the abnormal dilation of fetal cerebral ventricles due to the obstructed passage of cerebrospinal fluid, from its production in the choroid plexi to its absorption across the arachnoid villi [87]. Isolated aqueductal stenosis (IAS), which leads to non-communicating hydrocephalus by obstructing the CSF flow at the level of the aqueduct of Sylvius between the third and the fourth ventricle, i.e., the narrowest segment of the ventricular system, remains the most common cause of ISVM [88].

At present, postnatal CSF drainage achieved by the implantation of shunts or endoscopic third ventriculostomy (ETV) to bypass the occlusion and redirect the flow of the cerebrospinal fluid, remain the methods of choice for the management of congenital ISVM [56,89,90]. Postnatal surgical decompression of the swelling of cerebral tissue seems to be a delayed intervention because critically important stages of brain development occur in utero, and the consequences for the brain tissue, neuron migration, and molecular changes become irreversible, resulting in the deteriorating motor and neurocognitive function in the affected patients [91]. Postnatal interventions reverse the neurological deficits only partially and alleviate the symptoms by lowering the intracranial pressure [91,92]. Additionally, postnatal shunting is associated with a high incidence of shunt malfunction, estimated at 30% in the first year since implantation and 10% per year in the subsequent years of life [77]. Also, it is linked with an elevated mortality risk due to the delayed diagnosis of shunt malfunction [93,94].

ISVM is a cause of increased intracranial and/or intraventricular pressure, with hemodynamic and metabolic changes in the brain tissue. Glick et al. demonstrated that elevated CSF pressure causes decreased cerebral blood flow, regional ischemia, and metabolic changes in the periventricular axons [95]. Obeidat et al. investigated the impact of the abnormal MCA Doppler (absent or reversed diastole) in fetuses with VM and observed a statistically significant correlation between cerebral circulation abnormalities caused by elevated intracranial pressure and high perinatal mortality [96].

Destructive effects of SVM on the brain have been demonstrated on histopathology. Pathological changes include ependymal volume loss, disruption of the germinal matrix with destruction and glial changes in the periventricular axons and secondary neuronal loss, axonal degeneration with neuronal loss, and a decrease in synaptic density [97]. Abnormal myelination may also be the result of hypoxia and/or the effect of toxins from the cerebrospinal fluid [98].

Several authors described the evolution of AD value in IVM as a highly individual, long-term observation that requires repeated ultrasound testing. Rault et al., in their study of pediatric patients, described three patterns of prenatal imaging of IAS: rapid progression in the third trimester with abnormal white matter changes on MRI with the need for timely VP shunting, progressive ventriculomegaly in the second and third trimester with VP shunting delayed until year 1 of neonatal life, and stable ventriculomegaly with no need of postnatal intervention [21]. Perlman et al. compared pre- and postnatal imaging in a cohort of 92 fetuses with mild-to-moderate IVM (AD ≥ 10–<14 mm) and found a statistically significant decrease in ventricular dilation (*p* < 001). On clinical follow-up at 24 months, all children were normal except three, who presented with very mild neurological deficits [19].

In contrast, fetuses with ISVM with an AD of ≥15 mm identified at any gestational age may present with stable, progressive, or decreased AD values [19]. The dynamics of the ISVM development does not follow a single pattern.

These studies provide information that helps to identify cases that might benefit from the prenatal decompression of ISVM, as well as patients in need of urgent postnatal intervention [19,24,25].

## 5. The Result of Destruction Caused by Fetal ISVM

Scher MS, in “‘The First Thousand Days’ Define a Fetal/Neonatal Neurology Program”, reported that the cumulative result of neurological deficits caused by congenital defects of the central nervous system is the effect of the maternal/placental/fetal triad at the initial stages of gestation [99]. Developmental neuroplasticity of the cerebral tissue is more probable during the critically sensitive stages of brain maturation over the first 1000 days since conception [99]. ‘The First Thousand Days’ fetal/neonatal neurology program, by using the 1000-day observational perspective, clearly identifies the pregnancy-specific mechanisms affecting the maternal/placental/fetal triad, expressed as congenital cerebral abnormalities and destructive changes between second/third trimester and year 2 of life [100].

According to the SMFM Consult Series, mild or moderate IVM-related cortical damage is associated with low risk for postnatal neurosurgical intervention and a favorable neurodevelopmental outcome in 90% and 75–93% of the cases, respectively [16].

Several authors reported that changes in the cytoarchitecture may affect neuronal interaction and functionality, which may later be linked with schizophrenia, ADHA, and attention disorders [6,15,101].

In a new study conducted by Kyriakopoulou et al., nine out of twenty-four (37.5%) children with a prenatal diagnosis of ventriculomegaly met the criteria for autism spectrum disorder (ASD). A statistically significant correlation was found between ventriculomegaly and autism—ASD ADOS-2 (*p* = 0.024, chi2 = 5,1, df = 1). Neurodevelopmental studies of children with IVM with altered cortical development found ASD traits, such as difficulty to sustain attention, working memory, and cognitive behaviors [102]. MRI findings of fetal INSVM identify developmental age cohorts in need of long-term observation and follow-up [42].

### 5.1. SVM Decompression Before the IFMSS Moratorium

The initial attempts at prenatal SVM decompression via craniotomy in the last century aimed to decrease the fetal head circumference and facilitate vaginal delivery [103]. Likewise, repeated percutaneous ultrasound-guided evacuation of the fetal cerebrospinal fluid to lower the risk for perinatal maternal morbidity and mortality, without the therapeutic benefits for the fetus or neonate, has been described [104].

The development of ultrasound-guided neuro-scanning in the 1970s of the 20th century and the possibility of repeated observations, indicating a progressive cerebral degeneration in a fetus with SVM, led to the concept of permanent ventricular shunting [105]. Harrison et al. introduced the concept of single implantation of a one-way VAS system or an extended system of programmable pressure valves [105].

In 1982, Clewell et al. described a case of an ultrasound-guided percutaneous implementation of a shunt constructed of medical-grade Silastic tubing with the one-way valve in a patient with fetal hydrocephalus [106]. Early attempts at ultrasound-guided percutaneous VA shunting unfortunately resulted in 10% perioperative mortality and moderate-to-severe disability in 66% of the survivors [107]. In 1982, the International Fetal Medicine and Surgery Society (IFMSS) established a voluntary International Fetal Treatment Surgery Registry [108]. Between 1982 and 1985, 39 cases of in utero repair were registered, with the overall survival rate of 83% and procedure-related mortality of 17%. Over half of the patients presented with severe neurological deficits, 12% with mild or moderate deficits, and only 35% without developmental delays [99]. Poor clinical neurodevelopmental results of patients who survived in utero shunting, and higher mortality rates in those with postnatal shunt implementation, were the rationale behind the 1986 IFSSM voluntary moratorium against VAS until more compelling evidence of shunting efficiency was obtained [99,109].

### 5.2. Post-Moratorium Era—Advancements in Bio-Engineering and ISVM Decompression Techniques

Advancements in biomaterial engineering for VAS technology are aimed at manufacturing a non-immunogenic shunt, which will not be prone to clogging, dislocation, or infection [110]. The systems that have been used so far, i.e., Orbis-Sigma and Accu-Flow valves and Cook’s shunts, meet the conditions of unidirectional flow of the CSF and are equipped with a system that prevents overdrainage. However, due to shunt dislocation, they required frequent revisions, which were associated with preterm labor [107,110].

In 2016, Chen et al. introduced a shunt device for treating fetal aqueductal stenosis fabricated out of 3Fr or 4Fr catheters, which are characterized by longitudinal bending stiffness with kink resistance, a sufficient luminal area for cerebrospinal fluid drainage, and capacity for valve integration [111]. Emery et al. described experimental improvements to a VA shunt that contains a main silicone–nitinol composite tube, a super-elastic 90° angled dual dumbbell anchor, and an ePTFE valve encased by a stainless-steel cage [112]. The anchor changes its diameter, ranging from 1.15 mm in a collapsed state to 2.75 mm in a deployed state, demonstrating an up to 1.4-fold diameter change in human body temperature, which prevents shunt displacement [112]. For now, the functionality of these devices, which decrease the elevated intracranial pressure and prevent shunt migration as well as CSF reflux, is investigated in pre-clinical trials [113,114].

Voluntary ‘IFMSS moratorium’ against prenatal ventricular decompression in fetuses with SVM, which had been in force for thirty years, did not lead to the complete abandonment of such interventions [56].

Cavalheiro et al. described 39 fetuses with SVM, between 24 and 32 weeks of GA, who underwent intrauterine therapy [114]. VAS implementation was performed in 19 patients using the pigtail KCH-Rocket system. Postnatally, almost all patients (38/39) required VP shunting with low-pressure valves. These authors reported a total of 57 SVM decompressions using the following techniques: repeated cephalocentesis, VAS, and one successful endoscopic third ventriculostomy (EVT) [114]. Neurodevelopmental findings at year 3 of follow-up revealed normal development in 66%, moderate delay in 15%, and severe delay in 19% of the cases [114].

Fetal therapy specialists from the Institute of the Polish Mother’s Heath Center in Łódz, Poland, have performed shunting in 222 fetuses with SVM since 1992 [115]. The learning curve for that center for 2006–2012 demonstrates a 50% reduction in the AD values, with subsequent enlargement of the ventricular width in 25% of the cases. Complications were observed in 22 of the VAS patients, including four cases of intrauterine fetal demise at the mean gestational age of 34 weeks at birth. Almost 18% of the neonates did not require further neurosurgical interventions. Favorable neurological outcomes were found in 60% of the children [115].

The same center of fetal surgery published their results of 44 VAS interventions performed at a mean GA of 25 weeks, with an AD of >20, and confirmed isolated SVM [115]. These authors reported the following: three deaths after shunt insertion, 50%—shunt (Reusable Introducer Set; Rocket Medical plc, Washington, UK) dislocation to the amniotic cavity or the ventricle. Nevertheless, they also reported 41 live births at a mean GA of 37 weeks. A neurodevelopment assessment of the survivors with ISVM at year 2 of age was performed using the Bayley scale and classified as follows: nineteen (70.4%; 95% CI: 51.5–84.2%)—normal development or mild delay, eight (29.6%; 95% CI: 15.6–48.5%)—moderate or severe delay. In turn, out of eleven children with NSVM, normal development or mild delay was found in two (18.2%; 95% CI: 5.1–4.8%), and moderate or severe delay in nine (81.8%; 95% CI: 52.3–94.9%) cases [115].

## 6. Experimental and Clinical Results of ISVM Decompression

### 6.1. Endoscopic Third Ventriculostomy

Endoscopic third ventriculostomy (ETV) is performed by creating artificial communication between the floor of the third ventricle and the subarachnoid space, which allows for the cerebrospinal fluid to be evacuated, and normal intracranial pressure to be restored. Prenatal ETV is a percutaneous, minimally invasive procedure that lowers the risk for infection associated with shunt implantation or malfunction and prevents contact between the CSF and AF [116].

Studies on animal models with induced aqueductal stenosis, which determined the viability of neuroendoscopic (EVT) decompression through the foramen of Monro to achieve opening of the third ventricle, offered a novel approach to ventriculomegaly management in-utero.

Peiro et al. induced prenatal hydrocephalus in 50 ovine models by injecting a polymeric agent (E85, E105) into the third ventricle [117]. The endoscope was inserted in the point located anteriorly to the coronal suture, 7 mm from the midline; the floor of the third ventricle was identified in 64% (32/50) of the fetuses. In 36% of the cases, the authors reported obstacles caused by the presence of the polymer in the cerebrospinal fluid or choroid plexus bleeding. Successful puncture of the third ventricle floor by ETV has been accomplished in 80% (32/40) of ovine models [118].

Feasibility of prenatal EVT in ISVM drainage was confirmed by clinical studies of 11 cases of human fetuses who underwent surgery for ventriculomegaly [114,118,119,120]. Two clinical studies published their results of prenatal ETV.

**Figure 3:**
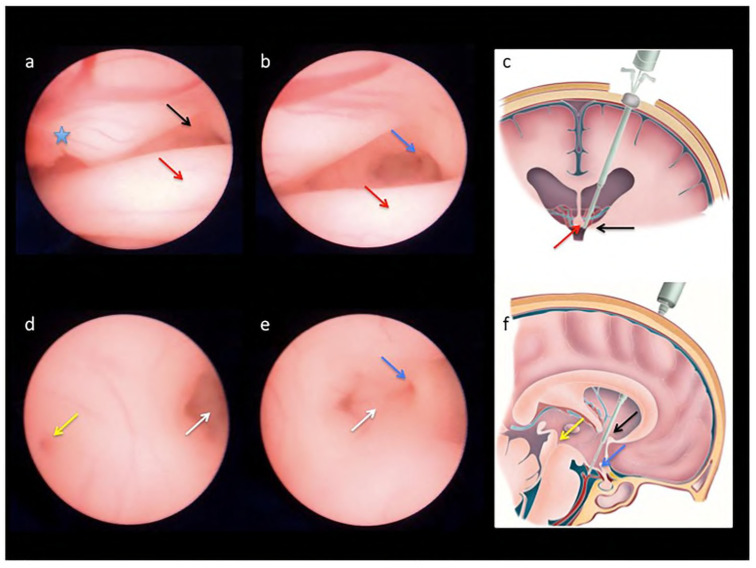
Endoscopic views obtained during percutaneous fetal third ventriculostomy in a case of severe ventriculomegaly (**a**–**e**) and schematic drawings of normal brain anatomy (**c**,**f**) to demonstrate the landmarks followed to reach the base of the third ventricle during the procedure: the anterior portion of the choroid plexus ((**a**); blue star), the foramen of Monroe ((**a**,**c**,**f**); black arrows) and the anterior column of the fornix ((**a**–**c**); red arrows), the infundibular recess ((**b**,**e**,**f**); blue arrows), the mammillary bodies ((**d**,**e**); white arrows), and the beginning of the cerebral aqueduct ((**d**,**f**); yellow arrows) [117].

In 2003, Cavalheiro et al. published the first case of endoscopic third ventriculostomy performed prenatally using an ultrasound-guided percutaneous technique [114]. A 2.5 mm trocar was introduced into through the anterior fontanelle to the lateral ventricle, as well as a 2.3 mm neuroendoscope with a 1 mm working channel (Neuroview, flexible scope, 25C, Traatek, Fort Lauderdale, FL, USA). The Fogarty 2Fr catheter was introduced into the third ventricle through the foramen of Monro and the membrane of Liliequist, achieving CSF evacuation. The fetus was delivered at 36 weeks of gestation by cesarean section [114]. In 2023, Peralta et al. reported 10 cases of ETV by percutaneous approach in human fetuses [120]. Cases with progressive, apparently isolated SVM with a mean GA of 28.7 weeks (25.3–30.7) were scheduled for surgery. As a result 7/10 fetuses that underwent the procedure had stabilized or decreased lateral ventricle atria diameter. The mean GA at birth was 38.2 weeks (35.9–39.3). EVT was performed using a 2.4 mm two-channel straight sharp-tip sheath (11530 KA, Karl Storz, Tuttlingen, Germany), a 1.2 mm semirigid endoscope (11540 AA, Karl Storz, Germany), and a 2F-Fogarty balloon catheter (Edwards Lifesciences, Kanada). The anterior horn of the lateral ventricle was reached by sharp sheath through the lateral vertex of the bregmatic fontanelle. Intraventricular pressure and intraamniotic pressure was measured. The Fogarty catheter was used to perforate the floor of the third ventricle, the balloon was filled with 0.2 mL of saline to create a suitable aperture. Post natal observations shown that, 80% of the neonates needed a VPS. Patients with AD values stabilized post prenatal ETV shown more favorable neurodevelopmental results, evaluated with the ASQ-3 test, as compared to the group with elevated values—(4/7) 57.1% vs. (3/3) 100%, respectively. Further research are needed to assess whether the earlier ETV treatment at 27 weeks of GA with a lower AD ISVM value could potentially improve the developmental outcome for these patients. The findings of Peralta et al., who proved the viability of this minimally invasive technique, mark a milestone in the development of ISVM neurosurgery. An illustrative comparison of normal fetal brain anatomy, vs. ventriculomegaly, and images obtained during ETV are shown in Figure 3.

At present, ETV is a commonly used method of VM decompression among pediatric patients [117]. Analysis of the results showed that the ETV and VPS both remain feasible treatment options for fetal hydrocephalus and have comparable success rates when used as first-line treatment [117].

EVT performed during the neonatal period helped avoid chronic shunting in some groups of patients [117,121]. The International Infant Hydrocephalus Study (IIHS) reported favorable outcomes for health and the quality of life in a cohort of patients who underwent ETV as their first treatment [122,123].

### 6.2. Open Fetal Surgery in Ventri Culo-Amniotic Drainage Isvm

The concept of using hysterotomy to implant the shunt for fetal SVM was previously suggested in 1986 by Michejda et al., who hypothesized that it might be a more effective method of ventriculo-peritoneal shunt implantation and could also become the treatment of choice in cases of eligible progressive hydrocephalus [123]. So far, 11 open fetal surgeries for shunt placement have been performed.

Al-Anazi et al. reported six cases of the ‘Al-Anazi VA’. Author team implanted a shunt system through a 1 cm incision in uterus. This was a one-way valve measuring 3 cm with a special winglets, that prevented its intra or extracranial displacement [124]. The initial case of system implantation, was performed at 32 weeks of GA. The fetus, despite reaching 37 weeks of gestation required shunt placement after birth due to overdrainage of the ventricles [124]. The following five pregnancies, were operated between 27 and 31 weeks of GA. Cerebrospinal fluid was successfully drained into the amniotic cavity along the pressure gradient through all shunts. Unfortunately, due to complications such as: placental ablation, shunt infection, septicemia, and severe developmental delay, further research has been discontinued [124,125].

In the next two studies, apart from the placement of a complex shunt system (a ventricular catheter, a one-way low-pressure valve), the possibility of postnatal distal shunt conversion to VPS was investigated [126,127].

Bruner et al. placed a shunt through a hysterotomy at 23.0–26.5 weeks of GA in four fetuses with obstructive aqueductal stenosis between 1999 and 2003 at the Vanderbilt University Medical Center. Their research was approved by the Institutional Review Board [126]. The eligibility process involved magnetic resonance imaging, repeated ultrasound measurements of the AD value—demonstrating a mean increase of 11.3 mm/week, genetic testing (karyotyping, aCGH), microbial profiling of the AF, genetic counselling, and prospective assessment of the child’s further development. Using Pfannenstiel incision the uterus was exteriorized. The shunting system included the following: a ventricular catheter and a low-pressure valve PS Medical Ultrasmall^®^ (Medtronic) were inserted with a needle using the Seldinger technique after making a 1 cm incision in the scalp above the right ear, in Keen’s point. The distal part of the catheter was subcutaneously tunneled to the exit near the fetal neck, with the possibility of VAS conversion to VPS after delivery. All deliveries occurred between 27 and 34 weeks of GA. All shunts performed well until delivery, which supports the feasibility of shunt placement and maintenance. Post-delivery, the distal drain was converted to a VP shunt in three patients. In three out of four neonate, infection was the main complication, ultimately resulting in the death of one of the newborns. Neurodevelopmental delay was unfortunately present in all these children [126].

The team of authors, Zamłyński et al. reported surgical ventriculo-amniotic valve implantation (VAVI) with open fetal surgery (OFS) performed at 24.4 weeks of GA [127]. As for the eligibility process for OFS, maternal exclusion criteria from the MOMS protocol included the following: cervical insufficiency and/or short cervix (<20 mm on ultrasound), a history of preterm labor (delivery at <37 weeks of GA), congenital Mullerian duct anomalies, postoperative deformity of uterus, type 1 diabetes, maternofetal Rh(D) isoimmunization, obesity defined as a BMI ≥ 35 kg/m^2^, positive TORCH screening, seropositivity for anti-HIV, HCV or HBV viremia, other significant comorbidities of a pregnant woman, psychosocial problems, the lack of a support system, and/or inability to travel and participate in the follow-up studies [128]. Fetal inclusion criteria were as followed: the diagnosis of ISVM on MRI—supratentorial hydrocephalus, without signs of CSF flow through cerebral hydrocephalus, normal karyotyping of amniotic fluid cells on aCGH, absence of other anatomical defects. Serial ultrasound measurements revealed progression of ventriculomegaly—AD value from 18.3 to 22.6—2 mm/7 days. 

**Figure 4:**
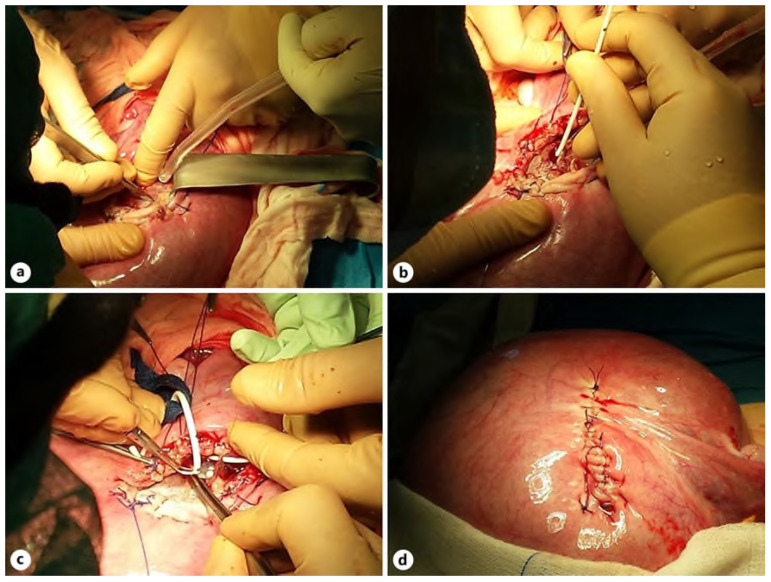
Stages of OFS for VAVI: hysterotomy, incision of the left temporal bone (**a**), implantation of the proximal VAVI part (**b**), implanted low-pressure valve, subcutaneous tunneling of the proximal part of the system toward the fetal nape (**c**), and myometrium after suture of the uterus (**d**) [127].

Pfannenstiel incision was used to open the abdominal cavity, uterus remained un-exteriorized. The team of authors followed and implemented the technique used by Bruner et al. [116]. The 46,220 Shunt Ultra Small 4CM LL (Medtronic PS Medical, Goleta, CA, USA) set was used. Further observations revealed an increase in parenchymal thickness and decompression of ISVM to AD value of 13.6 mm. Stages of OFS for VAVI are shown in Figure 4.

**Figure 5:**
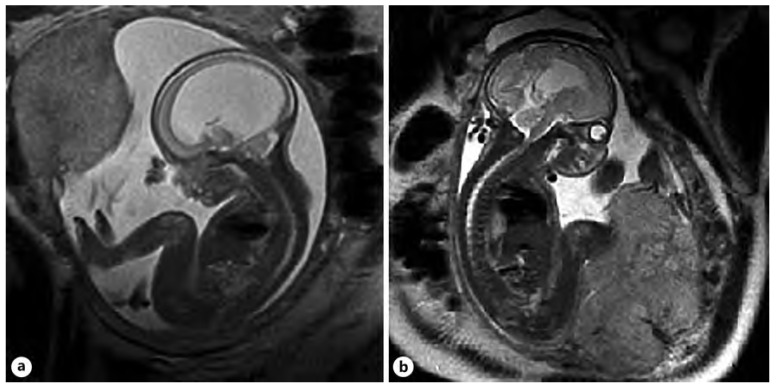
A comparison of the fetal MRI is shown in the sagittal plane, depicting the change in the lateral ventricular size of the brain pre- (**a**) versus post-VAVI (**b**) [127].

After delivery at 31.1 weeks of GA due to pPROM, the shunt was evacuated due to symptoms of infection. Later on, insertion of the Rickham reservoir and conversion to VPS resulted in a successful decompression of SVM. Follow up of patient at the age of 7 years old, revealed atypical autism and mild developmental delay. Additional genetic testing by WGS identified a variant in the *ZEB2* gene (NM_014795.4(ZEB2)):c.2813C> [127]. A comparison of the fetal MRI before and after OFS VAVI are presented on Figure 5.

## 7. Expected Potential of Fetal Surgery Center for Treatment of Fetal VM

The ‘IFMSS moratorium’, which has been in place for over 30 years—instituted in the absence of well-established paradigms of prenatal therapy and eligibility criteria for the procedure—markedly decreased the number of shunt implantations [121]. Still, potential ISVM candidates for prenatal shunt implantation should be diagnosed and hospitalized at the highest level of care specialist centers. The criteria for the highest level of care referral centers, in terms of NECESSARY RESOURCES for fetoscopic interventions requiring maternal laparotomy, have been recently published in the Care Levels for Fetal Therapy Centers study [129]. Likewise, Moldenhauer et al., in their recommendations for the highest level of care referral centers for fetal surgery, emphasized the need for accreditation and well-documented proof of the positive learning curve [130]. However, the recommendations in various publications did not include the VPS implantation techniques that might be applicable in ISVM therapy. The number of maternofetal surgery (MFS) centers that offer ‘shunting’ for ISVM must be limited to the national or regional level due to the complexity of the diagnostic process, the low prevalence of ISVM, and the required competence and expertise of the team. Accreditation and public reporting of the outcomes are a necessary requirement. The advantages and limitations of ISVM decompression techniques are presented in Table 1.

### 7.1. Model Candidates for Maternal–Fetal Surgery in the Treatment of ISVM

Maternal-fetal surgery (MFS) is a unique, sophisticated surgical procedure that involves both the mother and the fetus. Possible complications for the mother have to be carefully analyzed by an internal medicine specialist and anesthesiologist. Any maternal concerns about her condition and the rationale for the procedure should be addressed with psychological and/or ecumenical support [42,45]. The process of eligibility for MFS in ISVM for drainage system implantation via OFS should follow the MOMS protocol criteria [128]. Surgical and perinatal complications should be reported using the standard systems of Clavien–Dindo and the WHO [131,132].

An ISVM affected fetus is the actual MFS patient, with a dedicated VAS procedure. Careful selection and an adequate eligibility process increase the probability of a favorable outcome. Advanced repeated ultrasound testing, which would indicate a dynamic increase in AD values—2 mm/7 days to >20 mm, with degenerative thinning of the cortical mantle, is required [14,15,19,21]. Concomitant non-CNS and CNS-related defects should be ruled out with the use of US and MR imaging combined. Amniotic fluid testing by PCR is performed in the case of inconclusive test results of the suspected infection. The range of genetic testing should include amniotic fluid cell karyotyping with aCGH. Advanced WE or WG sequencing may be recommended for patients with suspected additional anomalies or complicated familial medical history [61,67]. During counseling, the parents of a child with ISVM should be informed that the percentage of survival rate without disabilities of affected children is 37% [85]. Finally, the mother should be made aware about the possibility of additional diagnoses later in life, even in the case of the most apparently isolated SVM.

### 7.2. Optimal Timing Window for ISVM Drainage

Knowledge about the natural history of fetal ventriculomegaly and the findings of trials on animal models define two clinical and pathomorphological stages of the disease, which determine the optimal time for the shunting intervention.

At the first stage of fetal hydrocephalus, supratentorial rupture, periventricular white matter, and axonal swelling are observed. The second stage is characterized by irreversible gliosis and demyelination [133,134]. If that process is stopped by a ventricular shunt in the initial stages of ependymal rupture, complete regeneration of the cerebral tissue may occur, with excellent fetal outcomes. However, if the same therapy was implemented in the second phase—when gliosis and demyelination are manifested—the outcomes might be disappointing [134].

The results of treating kaolin-induced ventriculomegaly with shunt implantation or VAS inserted through hysterotomy increased the rates of normal development and survival as compared to expectant management [85,134,135]. All authors agree that earlier intervention benefits the fetus as far as motor and cognitive development is concerned [134,135]. Based on this research, Sutton et al., in 2001, reported that fetuses with progressive ventricular dilation and cortical thinning before 28 weeks of GA might be more eligible candidates due to the fact that cerebral damage after 32 weeks of GA is most probably irreversible [135]. However, due to the progressive destruction of CNS cytoarchitecture by SVM and the achievement of diagnostic certainty of the isolated SVM type on US/MRI at 24 weeks of GA, Emery et al. proposed that this time should be considered the most appropriate when considering in utero drainage intervention [45].

## 8. Conclusions

### 8.1. Current State of Knowledge About Fetal ISVM

Accessibility of ultrasound imaging allows for an early diagnosis (since 18 weeks of GA) of fetal ventriculomegaly. Dedicated repeated neurosonography and advanced MRI identify non-severe (AD 10.0–14.9 mm) and severe (AD ≥ 15 mm) ventriculomegaly. The isolated nature of VM may be diagnosed only after excluding additional developmental intra- and extracranial defects and, with the help of genetic testing from chorionic villous sampling, amniotic fluid karyotyping and aCGH. ES-WES and WGS are a source of considerable knowledge, but the correlation of prenatal genotypes and postnatal phenotypes requires further studies. Active infectious agents for TORCH should be excluded.

Counseling for non-severe isolated ventriculomegaly should emphasize the overall favorable outcome for afflicted children with a low risk for neurosurgical intervention later in life. Long-term follow-up for CNS development using standard neurological tests is necessary. Severe isolated VM with the progression of AD values and a fetal head circumference of >95th percentile is correlated with high perinatal mortality rates. The prognosis for further neurodevelopment remains poor.

Counseling for all patients with VM should emphasize the transient nature of even the most apparently isolated type of VM. The parents should be made aware that further development of their child is likely to be associated with additional diagnoses, including ADHD and autism spectrum disorders. The option of TOP should be mentioned in non directive manner.

### 8.2. Future Research

Despite relatively favorable developmental findings in children with ISVM who underwent postnatal ventricular drainage, antenatal surgery is a delayed intervention. All changes associated with the VM-related damage—anatomicopathological, histologic, and functional—already take place in the prenatal phase of development. The relevance of the 30-year-old concept of voluntary moratorium should be revisited in light of the advancements in the fields of the diagnosis, bioengineering materials, and new surgical techniques of prenatal ISVM decompression. At present, the criteria for identifying ISVM seem to be well defined.

Further studies should focus on identifying ISVM candidates for prenatal ventricular decompression, including determination of the optimal gestational age, the cut-off point for ventricular dilation, the tempo of AD increase, and the thickness of the cortical mantle.

The future decompression technique of choice should be associated with a low level of surgical risk for the mother and stable, effective decompression with a shunting system, without overdrainage. There is a distinct need to determine the techniques of one-stage surgery, with the one-time prenatal implantation of a ventriculo-valvular system, with the possibility of distal shunt conversion to the peritoneal cavity. As far as the intraperitoneal CFS drainage is concerned, ETV seems to be the most promising technique.

### 8.3. Further Challenges

An RCT is the primary technique for objectively determining the results of a new method introduced. The design of the study must specify the diagnoses according to MedDRA terminology. Results should be reported only in standardized form, according to WHO parameters and the mandatory assessment of maternal morbidity in the Clavien–Dindo classification (CCI).

#### 8.3.1. Standardization of SVM Diagnosis

The diagnosis of NSVM or SVM should be based on complementary ultrasound/MRI techniques. Imaging provides information on the complete fetal anatomy. Not all malformations result in the exclusion of insularity. Abnormalities in the structure of the CC are often an accompanying lesion of SVM. The spectrum of genetic diagnostics should be limited and should address aCGH. WES tests often have uncertain significance and a claimed genotype–phenotype match. It is suggested that exome sequence testing should be reserved for patients with a burdened family history.

#### 8.3.2. Challenges of Counseling

Only a full panel of imaging, serial VM measurements matched with genetic, infectious tests, and family anamnesis are the basis for team-reliable counseling. NSVM has a counseling pathway.

The challenge in SVM counseling is to communicate the diagnostic data obtained to prospective parents and support persons. The counseling team should always point out possible concerns about the isolated nature of SVM. The purpose of the offered prenatal ISVM decompression procedure must be understood by patients.

#### 8.3.3. Challenges for FSC Centers Conducting RCTs

Due to the low prevalence of ISVM, the number of FSC centers should be limited to those that can conduct RCTs in the largest population area of pregnant women. The extent of ethical concerns should be determined by a decision of the local Bioethics Committee.

The FSC should offer the full spectrum:

- Diagnostics and counseling.
- Specialized staff and the technical possibility of performing ISVM decompression.
- Control of the neurodevelopment of children who have undergone prenatal ISVM decompression.

All FSCs should follow a uniform RCT protocol. All global results should be reported to the supervising unit.

## Figures and Tables

**Table 1:** The advantages and limitations of ISVM decompression techniques.

	Advantages	Limitations
Prenatal percutaneous EVT [Peralta]	minimally invasive percutaneous surgery ‘one-step’ surgical approach during pregnancy effective CSF drainage within the CNS during pregnancy no foreign body in the third ventricle—low risk for fetal infection one-port surgery low risk for maternal infection	challenging to position the fetal head challenging to locate the Monro foramen bleeding from the choroid plexus dedicated fetoscopic instrumentation the need for shunting using alternative technique during MFS need for postnatal shunting MFS complications: not reported
VAVI per OFS [Bruner, Zamłyński]	‘one-step’ surgical approach permanent VAVI implant without dislocation positioning of the fetal head and the Keen’s point is possible satisfactory control of the operative field effective CSF drainage during pregnancy possibility of VAS conversion to VPS without the need for valve removal	highly invasive maternal eligibility process using the MOMS exclusion criteria is required complex anesthesiologic procedures uterus must be exteriorized MFS complications: PROM, CAS, PTL
Ultrasound-guided VAS [Litwińska]	moderately invasive percutaneous intervention permanent one-way drainage low risk for infection	risk for several VAS reimplantation VAS dislocation to the amniotic cavity or the ventricle need for postnatal VP shunting MFS complications: IUFD, PTL, PROM

## Data Availability

All data available in this paper. No new data were created.
